# The role of protein lactylation in skin diseases: from molecular mechanisms to potential therapeutics

**DOI:** 10.3389/fimmu.2026.1853603

**Published:** 2026-07-01

**Authors:** Yue Zhang, Zhinan Shi, Xiaohui Mo, Qiang Ju, Zhanyan Pan

**Affiliations:** Department of Dermatology, Ren Ji Hospital, Shanghai Jiaotong University School of Medicine, Shanghai, China

**Keywords:** glycolysis, lactate, lactylation, post-translational modification, skin diseases

## Abstract

Lactylation represents a novel post-translational modification originating from lactate, a by-product of glycolysis. It has emerged as a pivotal mechanism linking cellular metabolism to epigenetic regulation. By transferring the lactyl group to lysine residues on both histone and non-histone proteins, this modification regulates gene transcription and protein function, thereby coordinating critical biological processes, including metabolic reprogramming and immune-inflammatory responses. Recent studies have highlighted the pivotal role of lactate and lactylation in the pathogenesis of diverse skin diseases, including immune-inflammatory skin diseases (e.g., psoriasis and atopic dermatitis), pathologic scars, skin malignancies (e.g., melanoma), and skin ageing. In certain diseases, such as psoriasis and melanoma, lactylation has been established as a core molecular mechanism, establishing a pathological link between systemic metabolic disorders, the local skin microenvironment, immune inflammatory processes, and skin diseases. In other conditions, lactate-mediated effects have been well documented, while the specific contribution of lactylation remains an active area of investigation. Currently, lactylation-related inhibitors have demonstrated therapeutic potential in cancer, metabolic, and immunological fields. Consequently, lactate-mediated lactylation may emerge as a novel therapeutic intervention target for skin disorders. In this review, we summarize recent advances in lactylation research in skin diseases and elucidate its potential as a therapeutic target for future clinical applications.

## 1 Introduction

Metabolic reprogramming constitutes a hallmark feature of pathological states across numerous diseases. Within this process, metabolites function not only as energy substrates but also as pivotal signalling molecules governing cellular fate. For a considerable period, lactate was regarded solely as a metabolic waste product arising from glycolysis under hypoxic conditions. However, an increasing number of studies indicate that lactate serves as both a significant energy source and a potent regulatory factor in biological processes, including immunomodulation and gene expression (1). In a seminal study in 2019, the team of Professor Zhao Yingming discovered a novel post-translational modification involving lysine lactylation, utilising lactate as its substrate. This modification directly links cellular metabolism to epigenetic regulation (2). Lactylation involves the transfer of a lactyl group from lactyl coenzyme A to a lysine residue on histone or non-histone proteins. This process is regulated by specific ‘Writers’ and ‘Erasers’ (3). This modification alters the physicochemical properties of proteins, thereby influencing chromatin structure, transcriptional activation, and enzymatic activity (4).

Accumulating evidence has indicated lactylation plays a pivotal role in diverse biological processes. Histone lactylation has been demonstrated to drive a ‘metabolite-epigenetic-neuroimmunological’ cascade, regulating macrophage/microglia polarisation and promoting angiogenesis (5, 6). Furthermore, by remodelling the tumour microenvironment (TME) and enhancing immune evasion, lactylation facilitates tumour progression and metastasis (7). As a highly active immunometabolic organ, the skin is particularly sensitive to systemic and local metabolic dysregulation. An increasing number of studies indicate that lactylation may play a role in the pathogenesis of multiple skin diseases, including autoimmune and inflammatory conditions (e.g., psoriasis, atopic dermatitis) (8, 9), fibroproliferative disorders (e.g., pathologic scars) (10), and skin malignancies (e.g., melanoma) (11, 12). This review aims to systematically summarise advances in research on lactate metabolism and lactylation in skin diseases, explore the potential mechanisms that drive disease progression, and investigate the clinical intervention prospects of metabolism and lactylation-targeted therapeutics. While lactate biology in skin disease is well-established, direct evidence for lactylation as a pathogenic driver remains emergent, representing a critical gap and opportunity for the field.

## 2 Lactate and lactylation

The primary pathway for lactate production within the body involves the conversion of glucose into pyruvate via glycolysis. Under anaerobic conditions, lactate dehydrogenase (LDH) then converts pyruvate into lactate. Cells and tissues primarily responsible for lactate production include red blood cells, hepatocytes, skeletal muscle cells, adipocytes, and skin tissues (13). Intracellular lactate can also be generated through glutamine catabolism (14), and lactate transport across the plasma membrane is primarily mediated by monocarboxylate transporters (MCTs) (15). In addition, lactate acts as a signalling molecule by binding to G protein−coupled receptor 81 (GPR81) (16, 17). For a long time, lactate was regarded as a metabolic by-product of glycolysis under hypoxic conditions. However, mounting evidence indicates that lactate can function as a signalling molecule or metabolic substrate, participating in energy metabolism, lipid metabolism, regulating redox reactions, and modulating post-translational modifications (PTMs) of proteins—particularly acetylation and lactylation modifications (18).

PTMs refer to covalent and enzymatic modification processes occurring after protein biosynthesis, representing critical steps in protein synthesis. Common PTMs include acetylation, methylation, phosphorylation, ubiquitination, SUMOylation, and ADP-ribosylation. By altering protein conformation, localisation, activity, and stability, PTMs regulate cellular biological processes. They are crucial for regulating protein function, protein interactions, cellular signalling, and gene expression, and are of significant importance in disease research. Over 650 types of protein modifications have been identified to date (19). Lactylation modification was first reported in 2019 by Professor Zhao Yingming’s team (2) as a novel post-translational modification (PTM). It involves the addition of a lactyl group onto lysine residues of histone or non-histone proteins. This process is typically catalysed by lactate transferases. Lactate reacts with the amino group of lysine via esterification or covalent bonding to form the lactylation modification. This modification significantly impacts gene expression, thereby influencing cellular metabolism, immune-inflammatory responses, and signal transduction.

Lactylation occurs extensively in both human histones and non-histones. These proteins are distributed throughout the nucleus and cytoplasm and directly regulate gene expression. Proteomic analyses have identified lactylation sites across diverse species (4), demonstrating their widespread occurrence in nature. However, the functional roles of these lactylation sites remain largely unknown and warrant further investigation. At the histone level, lactate-mediated lactylation primarily occurs on core histones (H2A, H2B, H3, H4) (20). Lactylation dynamically regulates DNA synthesis-related processes with high site-specificity. Functionally regulated sites currently include H2BK5la, H3K9la, H3K14la, H3K18la, H3K23la, H3K56la, H4K8la, H4K12la, and so on (2, 21, 22). The functional regulation of genes by these distinct sites differs significantly. For instance, H3K18la is associated with genes involved in immune inflammation and fibrosis (23–25). And H4K12la primarily targets genes involved in metabolic reprogramming and cell cycle regulation (26, 27). Beyond histone lactylation, non-histone lactylation also plays a crucial role, encompassing the lactylation of metabolic enzymes, transcriptional regulators, and signalling molecules. Non-histone lactylation influences cellular signalling pathways by regulating protein stability or activity (28).

## 3 Regulation of lactylation

Lactylation is a dynamic, reversible process whose levels are precisely regulated by local tissue and systemic lactate concentration and specific ‘Writers (lactyltransferases)’ and ‘Erasers (delactylases)’ (3). Research indicates that endogenous lactate produced by glycolysis may be a key regulatory factor in lactylation under both aerobic and anaerobic conditions. Exogenous lactate also exhibits a lactylation-promoting effect (2). Lactate concentrations undergo significant alterations during exercise, physiological stress, dietary variations (such as ketogenic diets), and metabolic disorders (e.g., impaired glycolysis, glucose metabolism, or fatty acid metabolism) (29, 30). Tissue hypoperfusion and anaerobic metabolism also markedly elevate lactate levels during inflammation or infection. Concurrently, inflammatory cytokines triggered by infection and inflammation can also cause elevated levels of lactate in both local tissues and the body (31, 32). Furthermore, within tumour tissues, cells exhibit a pronounced preference for glycolytic glucose utilisation for energy production even under oxygen-sufficient conditions, generating substantial lactate. This results in markedly elevated intracellular lactate concentrations relative to those in normal tissues. This phenomenon is known as the Warburg effect (33–35). Under pathological conditions, abnormal lactate concentrations arising from disrupted lactate metabolism drive abnormal lactylation, thereby promoting disease progression through the activation of oncogenic pathways (2, 9, 36, 37).

**Figure 1:**
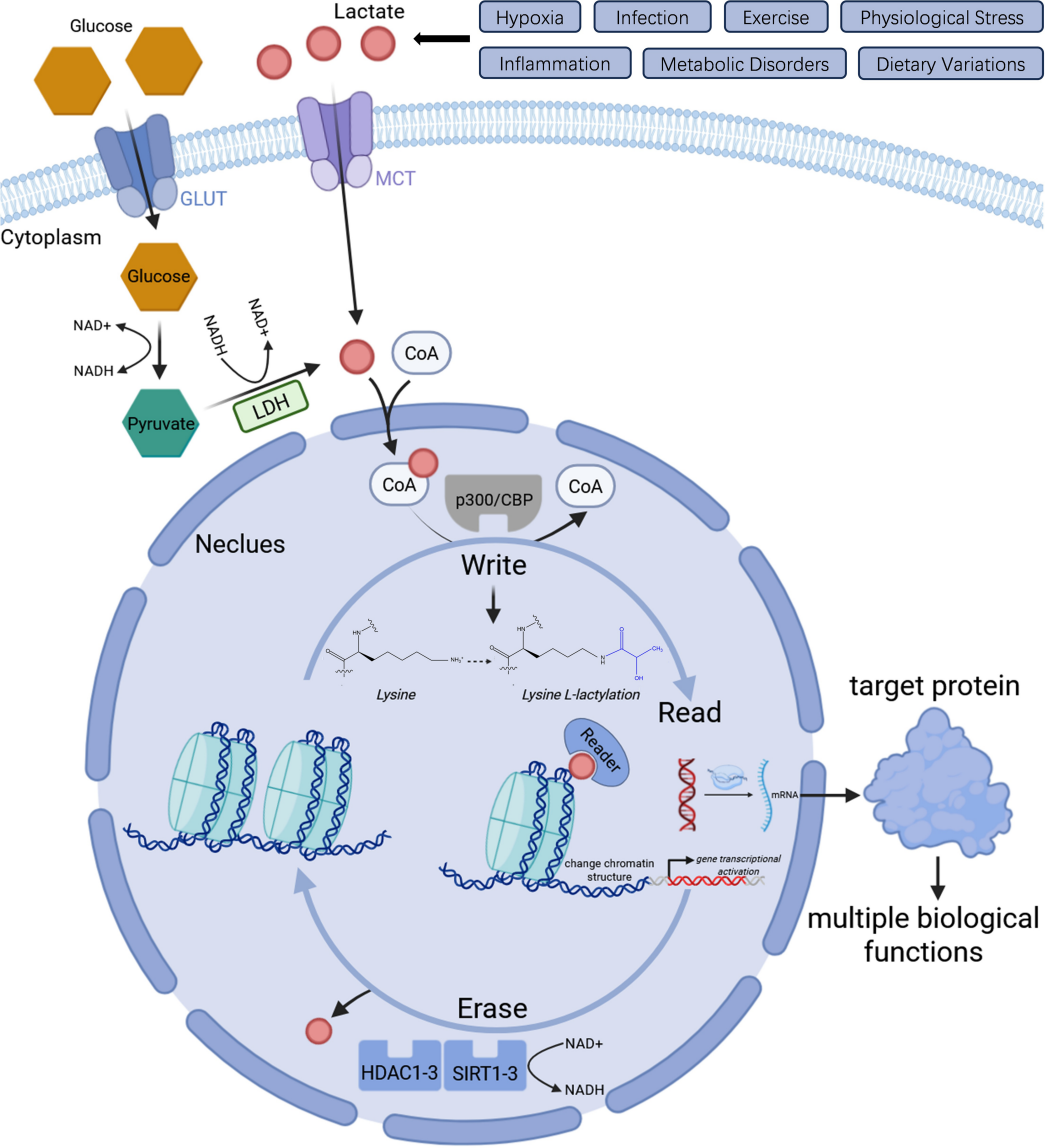
Lactate generation and regulatory mechanisms of Lactylation. This schematic illustrates the metabolic origins of lactate and its conversion into lactyl-coenzyme A (Lac-CoA) for protein lactylation. In the cytoplasm, lactate enters the cell via monocarborxylat transporter (MCT). And glucose enters the cell via glucose transporters (GLUT) and is metabolized through glycolysis to pyruvate. Under aerobic or anaerobic conditions, lactate dehydrogenase (LDH) converts pyruvate to lactate, with concurrent NADH oxidation to NAD^+^. Lactate is transported into the cell via MCT. Intracellular lactate is converted to Lac-CoA, which serves as the substrate for lactyltransferase enzymes. Lac-CoA then enters the ‘Write-Read-Erase’ cycle. 1) Write: The histone acetyltransferase family (p300/CBP) catalyzes the transfer of lactyl groups from Lac-CoA to the ϵ-amino group of lysine residues on histone or non-histone proteins, forming lysine L-lactylation. 2) Read: Lactylation readers recognize the modified lysine, inducing chromatin structural changes and transcriptional activation of target genes, ultimately producing mRNA and regulating multiple biological functions. 3) Erase: Histone deacetylases (HDAC1-3) and sirtuins (SIRT1-3) function as delactylases, removing lactyl groups in an NAD^+^-dependent or -independent manner to restore the unmodified state. GLUT, glucose transporter; MCT, monocarboxylate transporter; LDH, lactate dehydrogenase; CoA, coenzyme A; Lac-CoA, lactyl-coenzyme A; HDAC, histone deacetylase; SIRT, sirtuin; NAD^+^/NADH, oxidized/reduced nicotinamide adenine dinucleotide.

The mechanism by which lactate binds to lysine residues within cells during lactylation represents a core scientific question. Similar to other post-translational modifications, the regulation of lactylation can be divided into the following steps: 1) Lactate activation: Lactate combines with coenzyme A (CoA) to form lactyl-CoA (Lac-CoA). 2) Lactyl groups transfer: Lactyl groups are transferred from Lac-CoA to the ϵ-amino group of lysine residues on the target protein, catalysed by lactyltransferases, termed ‘Writers’. 3) Lactylation Recognition: Specific ‘Readers’ bind the lactyl groups, recognising the lactylation on the target protein and transmitting signals. Concurrently, they alter the tightness of histone-DNA binding, thereby indirectly regulating gene expression. The precise recognition mechanism remains under investigation. 4) Lactyl groups removal: Upon completion of modification, ‘Erasers’ catalyse the reversal of lactylation, removing lactyl groups from the lysine residue to restore the target protein to its original state (shown in **Figure 1**). In this process, the writers and erasers determine the reaction rate of lactylation. Changes in the quaternary structure of these enzymes affect their activity, ultimately influencing the lactylation level of the target protein (38, 39). Elucidating the identity and function of these enzymes is key to understanding the biological significance of lactylation.

### 3.1 ‘Writers’: lactyltransferases

The enzymatic addition of lactyl groups to lysine residues is primarily mediated by histone acetyltransferases (HATs) that utilize lactyl−CoA as a substrate (3, 40). p300 and its homologue CBP are well−recognized lactyltransferases, lactylating both histone (e.g., H3K18) and non−histone proteins (e.g., HMGB1, MRE11) (2, 41, 42). In skin diseases, writer activity displays marked cell-type specificity. In psoriasis, IL-17A induces keratinocyte-specific HAT1 to catalyse H4K12 lactylation, which upregulates KLK8 and IL-17R to form a self-amplifying inflammatory loop (8). In dermal fibroblasts, the lysine acetyltransferase KAT8 acts as a non-histone lactyltransferase that catalyses LTBP1 lactylation at K752, enhancing TGF-β signalling and type I/III collagen synthesis (43). Furthermore, p300/CBP-mediated lactylation has been directly implicated in psoriatic fibroblast activation; the p300/CBP inhibitor A485 normalises psoriatic fibroblast gene expression and reduces psoriasis-like skin inflammation *in vivo* (44), demonstrating that writers translate the hyperglycolytic state of specific cutaneous cells into disease-driving epigenetic programmes. Beyond p300/CBP, several other HATs have been identified as lactyltransferases. for example, KAT8 acts as a pan−lactyltransferase, modifying eEF1A2 to promote protein synthesis and tumorigenesis (45). HBO1 (KAT7) catalyses H3K9la at transcription start sites, influencing cancer−related gene expression (46). KAT2A (GCN5) directly lactylates histone H3 at K14 and K18 when complexed with phosphorylated ACSS2 (47). TIP60 (KAT5) mediates NBS1 lactylation at K388, contributing to DNA repair and chemoresistance (48). Additionally, alanyl−tRNA synthetases AARS1 and AARS2 have emerged as lactate sensors that directly use lactate to lactylate target proteins, including p53 and YAP (49, 50).

### 3.2 ‘Erasers’: delactylases

The reverse regulation of lactylation is primarily mediated by histone deacetylases (HDACs), which act in opposition to histone acetyltransferases by ‘erasing’ lactyl groups (3). To date, 18 HDACs have been identified, categorised into Zn²^+^-dependent (HDAC1–11 classified as Classes I, II, and IV) and NAD^+^-dependent (SIRT1-7, Class III) types (51–53). Among these, Class I (HDAC1-3) and Class III (SIRT1-3) HDACs represent the most potent delactylating enzymes *in vitro* (2). And HDAC1 and HDAC3 exhibit the strongest deacetylating activity (51).

In cutaneous pathophysiology, the writer-eraser balance is frequently disrupted. In psoriatic lesions, HDAC1 expression is significantly upregulated, whereas SIRT1 expression is markedly reduced in the basal epidermis (54). This eraser imbalance may contribute to aberrant histone lactylation accumulation and sustained keratinocyte hyperproliferation. Notably, HDAC3—a potent delactylase (51)—is markedly elevated in psoriasis lesions and imiquimod-induced models, where it promotes keratinocyte oxidative stress, mitochondrial DNA leakage, and cGAS-STING pathway activation, thereby driving cutaneous inflammation (55). These findings suggest that HDAC3 functions as a critical epigenetic-immunometabolic checkpoint in keratinocytes, and its delactylase activity may be overwhelmed by excessive lactate production under inflammatory conditions. In allergic skin inflammation, HDAC3 has been shown to regulate MCP1 expression and mediate mast cell-dependent anaphylaxis (56), further underscoring the broad role of erasers in cutaneous immunity.

Increasing evidence confirms that SIRT family members (Class III HDACs) play a pivotal role in mediating the reversal of lactylation. SIRT1 (primarily located in the nucleus), SIRT2 (primarily located in the cytoplasm), and SIRT3 (primarily located in the mitochondria) efficiently remove lactyl groups from both histones (e.g., H3K18la) and non-histones (57, 58). This process is NAD^+^-dependent, mirroring the mechanism of SIRT deacetylation. Acting as delactylases, SIRTs establish a critical metabolic checkpoint. When NAD^+^ levels are high (typically reflecting energy sufficiency or a low glycolytic state), SIRT activity increases, and lactylation level decreases. Conversely, when lactate concentrations rise and the NAD^+^/NADH ratio decreases, lactylation tends to accumulate. This enables lactylation to respond sensitively to changes in cellular energy and metabolic states.

## 4 Lactate and lactylation in skin physiological functions

In 1972, Johnson JA et al. (59) proposed the concept of the ‘skin Cori cycle’. This concept posits that lactate produced by skin cells can enter the bloodstream, be transported to the liver, and be converted into glucose, thereby supporting glucose metabolism in the body. Subsequently, Figlak et al. (60) demonstrated that human scalp hair follicles operate an internal Cori cycle, showing that outer root sheath keratinocytes significantly increase glycogen synthesis when incubated with lactate. As our review published in 2025 summarized, skin constitutes one of the primary sources of lactate within the body, revealing a significant association between lactate and skin homeostasis (61).

**Figure 2:**
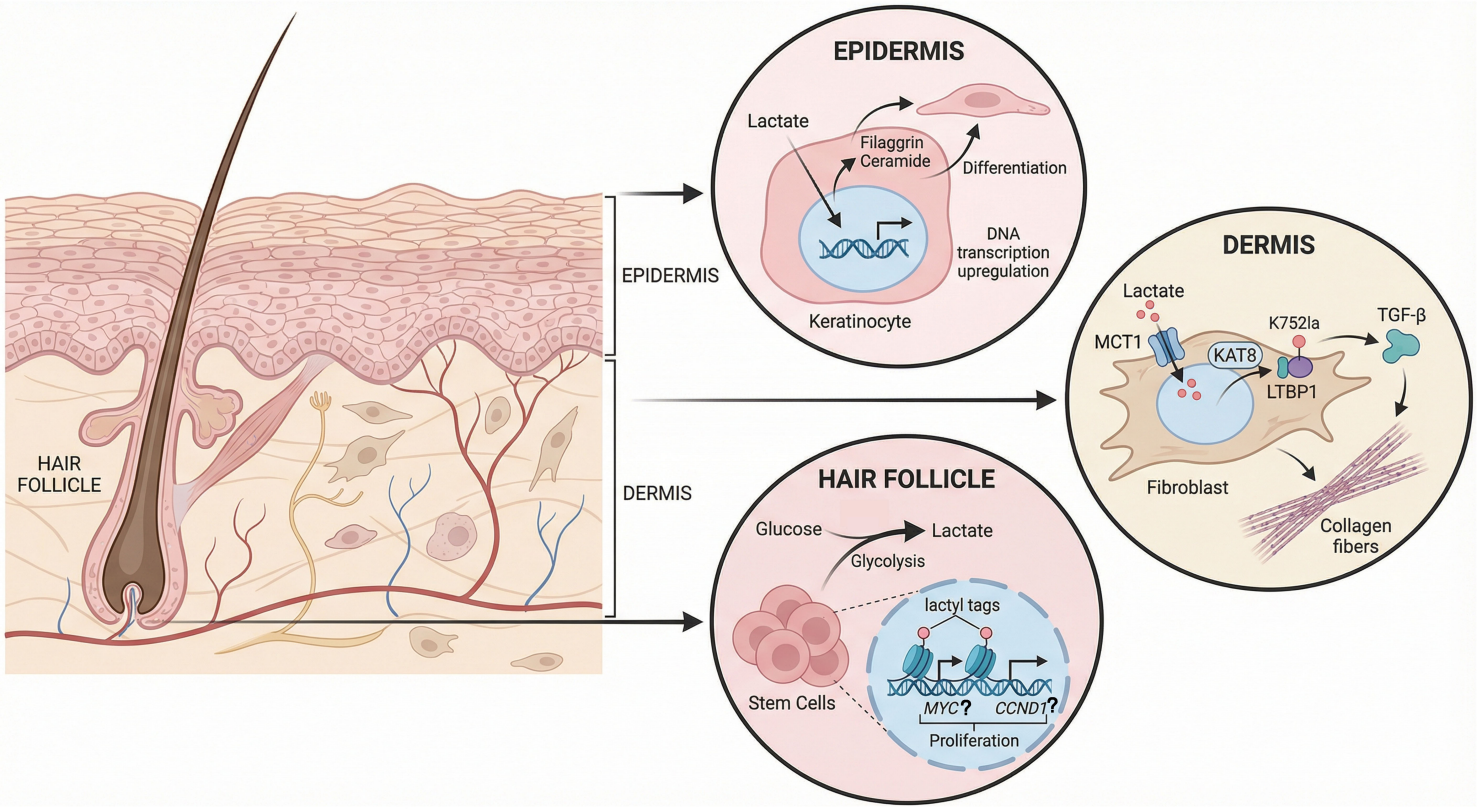
The role of lactylation in skin physiological processes. Three distinct skin compartments where lactate and lactylation regulate physiological functions are depicted. In epidermis, in keratinocytes, lactate promotes terminal differentiation by upregulating filaggrin and ceramide synthesis, core components of the skin barrier. Lactate-mediated signaling enhances DNA transcription of differentiation-related genes. The potential involvement of histone or non-histone lactylation in this transcriptional regulation is indicated as a hypothetical mechanism. In Dermis, In fibroblasts, lactate transported via monocarboxylate transporter 1 (MCT1) enters the nucleus and upregulates histone acetyltransferase KAT8 expression. KAT8 catalyzes lactylation at lysine 752 (K752la) of latent transforming growth factor-β binding protein 1 (LTBP1). This modification enhances LTBP1 binding to transforming growth factor-β1 (TGF-β1), promoting TGF-β signaling and subsequently increasing type I and type III collagen fibre synthesis. In hair follicle, hair follicle stem cells exhibit active glycolytic metabolism, converting glucose to lactate. Lactate may drive stem cell proliferation through histone lactylation, potentially activating proliferation-promoting genes such as MYC and CCND1 (indicated with question marks as hypothetical targets). This metabolic-epigenetic coupling may coordinate the anagen phase of the hair cycle. MCT1, monocarboxylate transporter 1; KAT8, lysine acetyltransferase 8; LTBP1, latent transforming growth factor-β binding protein 1; TGF-β1, transforming growth factor-beta 1; K752la, lactylation at lysine 752; CCND1, cyclin D1.

For a considerable period, the contribution of lactate to skin physiology has been attributed primarily to its role in maintaining the acidic surface environment of the skin, acting as a natural moisturising factor, and serving as a metabolic substrate (62–66). However, with the discovery of lactylation, the potential role of lactate as an epigenetic regulatory molecule is increasingly evident. Recent research indicates (8) that lactate can modulate the inflammatory cascade in immune-inflammatory skin diseases through protein lactylation, thereby influencing the efficacy of biologic therapies targeting specific inflammatory factors. This discovery highlights the pivotal role of lactate-mediated lactylation as a key molecular bridge linking systemic metabolism with epigenetic regulation in the pathogenesis of skin disorders. Previous studies have demonstrated that lactate plays a crucial role in fundamental physiological processes by regulating gene expression. These include maintaining skin barrier integrity, influencing skin cellular fate, coordinating cellular energy metabolism and biosynthesis, controlling hair follicular development and the hair cycle, and modulating skin immune cell function (shown in **Figure 2**). This hints that lactylation may also play a crucial role in these processes.

### 4.1 Maintaining epidermal homeostasis in skin

The terminal differentiation of the epidermis is a precisely regulated process involving the equilibrium of keratinocyte proliferation, differentiation, and apoptosis, which is crucial for the formation of the skin barrier. Lactate plays a crucial role in this process. Studies indicate that lactate can induce the expression of a series of differentiation markers in keratinocytes (e.g., filaggrin), and ceramide synthesis (67, 68). It remains unclear whether these effects are mediated by lactylation. Although lactylation can regulate gene expression in other cell types, there is currently a lack of evidence directly linking lactylation at specific sites to epidermal differentiation or barrier homeostasis. Therefore, while lactylation may be involved in these processes as a downstream effector of elevated lactate levels, this remains merely a hypothesis. Further research is needed to elucidate how lactylation functions in the epidermis.

### 4.2 Determining the fate of skin cells

The metabolic state of skin cells, such as fibroblasts and keratinocytes, is closely linked to their functional roles (69). Within keratinocytes, lactate contributes to keratinocyte apoptosis by modulating the expression of relevant proteins and enzymes (70). Furthermore, Baba H et al. (67) demonstrated that lactate helps upregulate mRNA expression of keratin 10 (an early differentiation marker) and involucrin (a late differentiation marker) in keratinocytes. It also enhances profilaggrin mRNA expression in a dose-dependent manner, promoting keratinocyte differentiation by increasing terminal epidermal differentiation. Salgaonkar N et al. (62) further confirmed that lactate enhances filaggrin expression, a marker of late keratinocyte differentiation. Cellular metabolic states can directly influence cellular fate decisions, with protein modifications playing a pivotal role (71). Lactylation, which bridges metabolism and epigenetics, directly senses fluctuations in the intracellular lactate level to determine cellular fate. Lactylation has been shown to directly regulate the proliferation and apoptosis of skin cells. In keratinocytes, histone H4K12 lactylation (H4K12la) drives IL-17A−mediated keratinocyte proliferation in psoriasis (8), and lactate−induced METTL3 upregulation via H3K18la promotes keratinocyte proliferation during wound healing (72). In dermal fibroblasts, H3K18 lactylation mediates lactate−induced fibroblast proliferation and collagen expression in keloids (73), and macrophage−derived lactate drives fibroblast phenotypic remodelling through H3K23 lactylation in hypertrophic scars (74). In melanoma cells, lactylation promotes cell proliferation, migration, and invasion (75). This collectively indicates that lactylation may exert a crucial regulatory role in determining the fate of skin cells. This process may occur by targeting post-translational modifications of proliferation/differentiation markers and apoptosis-associated proteins, directly modulating their activity and function to determine cellular fate. But this awaits experimental validation.

### 4.3 Coordinating the energy metabolism and biosynthesis of skin cells

Research indicates that multiple post-translational modifications (e.g., acetylation, phosphorylation) can regulate metabolic enzyme activity, thereby playing a pivotal role in controlling cellular energy metabolism (76–80). Notably, studies have revealed that lactylation can regulate intracellular glycolysis by modifying enzymes such as PKM2 and aldolase A (ALDOA). In LPS-induced macrophages, lactate enhances PKM2 activity through increased K62la of PKM2, thereby inhibiting glycolysis and promoting the transition of pro-inflammatory macrophages towards a reparative phenotype (81). Furthermore, Wan N et al. (82) demonstrated that K147la of ALDOA, a conserved metabolic enzyme across various human tumour cell lines, markedly reduced enzyme activity. Previously, lactate was thought to regulate glycolytic flux and its own biosynthetic pathway via non-covalent interactions (83). This discovery reveals a novel feedback regulatory mechanism for lactate-mediated glycolysis modulation: metabolic enzymes upstream of the glycolytic pathway undergo lactate-mediated lactylation, thereby inhibiting glycolytic activity following lactate accumulation. It may be inferred that a feedback pathway analogous to that observed in immune and tumour cells may also exist in skin cell metabolism. If operative in skin cells, lactylation could potentially regulate the glycolytic flux within skin cells by modulating the activity and expression of key metabolic enzymes such as PKM2 and LDHA, thereby adapting to varying energy metabolism and biosynthetic demands. However, direct demonstration of such a feedback loop in skin cells is currently lacking. Zou Y et al. (43) discovered that in dermal fibroblasts, the lactate released by poly-L-lactic acid (PLLA) degradation enters the nucleus via monocarboxylate transporter 1 (MCT1), thereby upregulating histone acetyltransferase KAT8 expression. KAT8 subsequently catalyses lactylation at the K752 site of latent transforming growth factor-β binding protein 1 (LTBP1). The K752la enhances LTBP1 binding to TGF-β1, thereby promoting TGF-β signalling and ultimately increasing the synthesis of type I and III collagen. This not only reveals the pivotal role of lactylation in regulating extracellular matrix synthesis and maintaining skin firmness and youthfulness, but also underscores the significant physiological function of non-histone lactylation in skin.

### 4.4 Regulating hair follicle development and the hair cycle

Hair undergoes an active cycle (anagen, catagen, telogen), a process dependent upon the precise activation of hair follicle stem cells and intense metabolic reorganisation. Research indicates that hair follicle stem cells exhibit robust glycolytic activity and lactate production upon activation (84). Lactate is a key signalling molecule that promotes hair follicle stem cell activation and hair growth (84–87). Moreover, an increasing number of studies confirm that lactate can modulate cancer progression by regulating the cell cycle (88). Liu S et al. (89) discovered that lactate-treated hepatocellular carcinoma (HCC) cells exhibited K249la of TPX2, a process regulated by lactate-binding protein (CBP) and histone deacetylase 1 (HDAC1). TPX2 lactylation is essential for cell cycle regulation and tumour growth. It disrupts the interaction between protein phosphatase 1 (PP1) and AURKA, thereby enhancing AURKA T288 phosphorylation and promoting cell cycle progression. This further demonstrates that lactylation plays a pivotal role in cell cycle regulation. Given the close relationship between the hair growth cycle and the cell cycle, and the established role of lactate in hair follicle, lactylation emerges as an attractive hypothetical mechanism for regulating the hair growth cycle. However, no studies to date have directly mapped lactylation sites or manipulated lactylation enzymatic machinery in hair follicle cells. For instance, specific histone lactylation may exist in anagen hair, driving rapid proliferation and differentiation of hair follicle stem cells by activating proliferation-promoting genes such as MYC and CCND1. Consequently, lactylation may serve as a crucial molecular switch coordinating the metabolic state of hair follicles with their cycle transcriptional programmes. This represents an important unanswered question at the intersection of metabolic epigenetics and skin biology.

### 4.5 Modulating the function of skin immune cells

As the largest immune organ in the body, the maintenance of immunological homeostasis in the skin relies upon the concerted action of epidermal cells and cutaneous immune cells. Immune cells, constituting a vital component of the cellular architecture in the skin, play a pivotal role in both the immune response to skin diseases and immunotherapeutic interventions. Lactate can mediate immune responses by regulating the metabolism of immune cells, thereby controlling their proliferation and functional activity (90). Lactate-mediated lactylation participates in immune signalling and inflammatory cascades by regulating the functions of key immune cells such as macrophages, mast cells, and T cells.

Macrophages, as one of the innate immune cells in the skin, can be categorised into two distinct phenotypes based on their functional roles: pro-inflammatory and anti-tumour M1 macrophages, and anti-inflammatory and pro-tumour M2 macrophages. Multiple studies have demonstrated that lactate can mediate epigenetic changes within macrophages through lactylation, regulating their transition between M1 and M2 phenotypes and thereby modulating the release of inflammation-associated factors (2, 91, 92). Furthermore, Yang K et al. (41) discovered that macrophages can uptake extracellular lactate via monocarboxylate transporters (MCTs), promoting lactate-mediated HMGB1 lactylation and release through a p300/CBP-dependent mechanism. As a widely distributed nucleoprotein, HMGB1 can be released by activated macrophages to coordinate inflammatory responses. These findings collectively underscore the pivotal role of lactylation in regulating macrophage function. By modulating the activity of inflammatory transcription factors, it balances pro-inflammatory mediators (e.g., TNF-α, IL-1β) against anti-inflammatory factors (e.g., IL-10), potentially influencing cutaneous inflammatory responses.

Mast cells participate in multiple skin inflammatory responses, simultaneously mediating allergic reactions, regulating inflammatory balance, and contributing to skin immune defence and tissue repair. Xu XT et al. (93) observed in IgE antigen-stimulated mouse bone marrow-derived mast cells that royal jelly acid inhibited intracellular H3la and H3K9la, leading to reduced histone lactyl-lysine enrichment at RELA and NFκB1 promoter sites, thereby suppressing IgE-dependent mast cell activation.

Lactylation can also influence T cell activation, proliferation, and cytokine secretion by regulating glycolytic metabolism (94). Lopez Krol A et al. (95) observed markedly elevated histone lactylation levels in lactate-treated CD4^+^ T cells, particularly Th17 cells. Following either CD3 monostimulation or combined CD3+CD28+IL-2 stimulation, lactylation levels in CD4^+^ T cells also markedly increased, indicating that histone lactylation is an intrinsic feature of CD4^+^ T cell activation. Activated T cell subsets (Th1, Th17, Treg) all exhibit high levels of histone H3la, suggesting widespread presence of this modification in activated T cells. Furthermore, the genome-wide distribution of H3K18la in lactate-treated Th17 cells predominantly enriched regions associated with T cell receptor (TCR) signalling pathways (including NF-κB and MAPK pathways) and the promoter region of Foxp3, the core transcription factor of Tregs. This indicates that lactate drives Th17 cell transcriptional reprogramming via lactylation, suppressing IL-17A secretion and inducing Foxp3 expression. This facilitates the conversion of pro-inflammatory Th17 cells into Treg-like cells with immunosuppressive functions, ultimately alleviating Th17-mediated intestinal inflammation *in vivo*. This underscores the role of lactylation as a key ‘metabolic-epigenetic’ molecular mechanism regulating T cell phenotype and inflammatory responses. Gu et al. (96) demonstrated that lactate promotes lactylation of MOESIN at K72, thereby influencing the maturation and immunosuppressive capacity of regulatory Treg cells. Furthermore, HCC patients responding to anti-PD-1 therapy exhibited reduced MOESIN lactylation levels in Treg cells compared to non-responders. These findings suggest lactylation serves as an epigenetic mechanism linking metabolic status to immune regulation. By modulating skin immune cell function and cytokine release, it may regulate local skin immune responses and contribute to the pathogenesis of skin diseases.

## 5 Lactylation in skin diseases

### 5.1 Psoriasis

Psoriasis is a chronic immune-inflammatory skin disease influenced by genetic, metabolic, and environmental factors. It primarily arises from abnormal activation of the cellular immune system and excessive proliferation of keratinocytes. This disease primarily involves Th17 cells and the IL-23/IL-17 axis, leading to overproduction of proinflammatory cytokines, including IL-17, IL-22, and TNF-α (97–99). These cytokines promote excessive keratinocyte proliferation, angiogenesis, and infiltration of immune inflammatory cells. Ultimately, they exacerbate skin lesions and systemic inflammatory responses. Research indicates a correlation between glycolysis and lactate metabolism levels and psoriasis severity (100). Serum metabolomic analysis reveals elevated lactate levels, derived from glycolysis, in patients with psoriasis compared with healthy controls (101). However, psoriasis patients frequently present with concomitant metabolic disorders, typically exhibiting elevated serum lactate levels. Consequently, whether the lactate detected in blood originates from psoriasis skin lesions or relates to psoriasis-associated metabolic conditions remains to be further investigated. Moreover, lactate is closely associated with persistent inflammation through its involvement in immune cell metabolic processes and functional regulation. Subudhi et al. (102) discovered that IL-17 activates glycolysis in epidermal cells via HIF1-α, thereby enhancing γδT17 responses through lactate. This increases IL-17 secretion and further intensifies inflammatory reactions. This indicates that lactate may indirectly amplify psoriatic inflammation by modulating immune cell differentiation and function.

**Figure 3:**
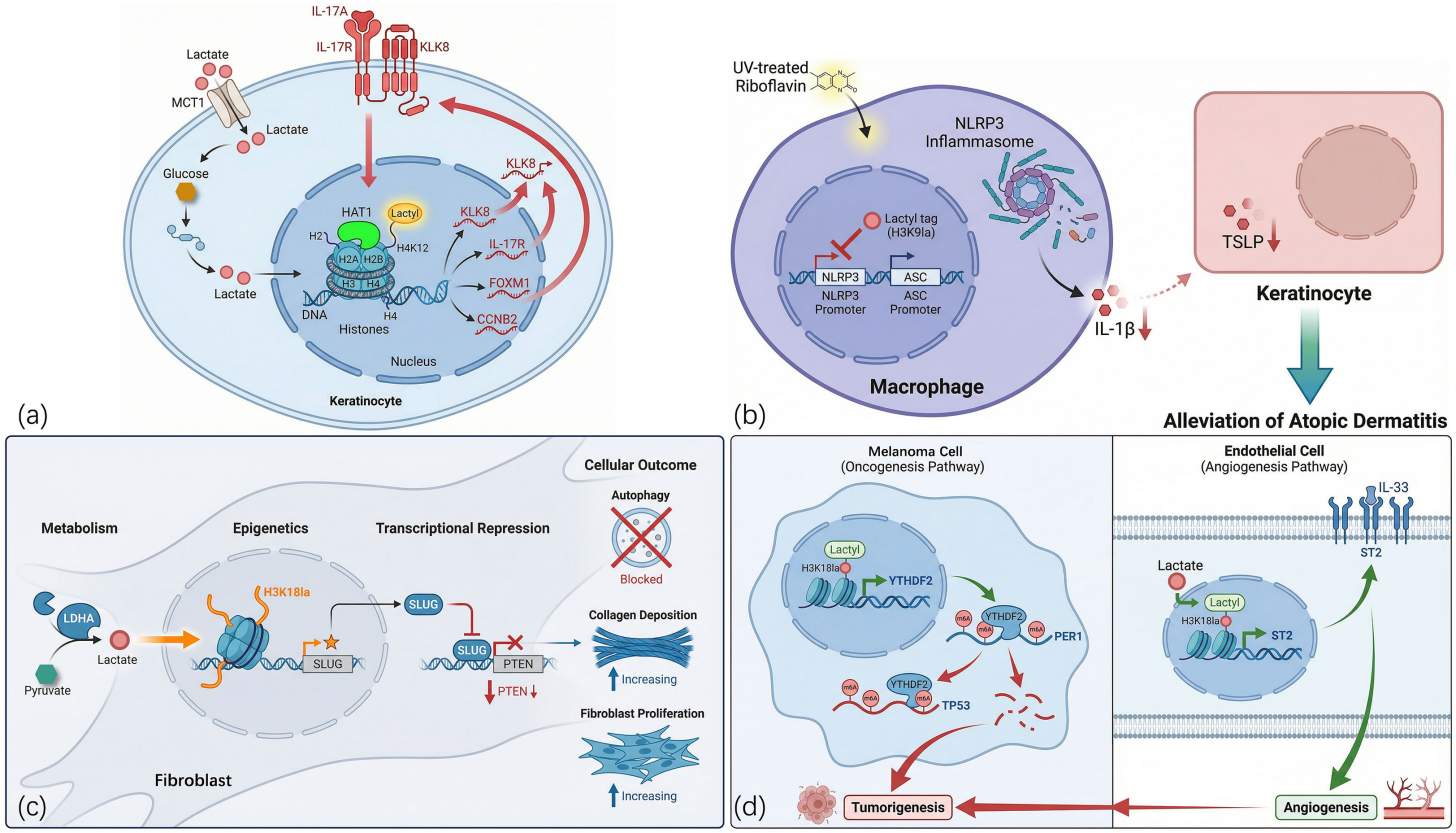
The role of lactylation in skin diseases. **(A)** Psoriasis: In keratinocytes, interleukin-17A (IL-17A) induces keratinocyte-specific kallikrein 8 (KLK8) expression, which interacts with the IL-17 receptor (IL-17R). This interaction promotes histone acetyltransferase 1 (HAT1)-mediated histone H4 lysine 12 lactylation (H4K12la). H4K12la upregulates KLK8, IL-17R, and cell cycle-associated genes (FOXM1, CCNB2, CDC25C), accelerating keratinocyte proliferation and forming a positive feedback loop. Elevated lactate in the microenvironment, transported via monocarboxylate transporter 1 (MCT1), drives this atypical IL-17A signaling pathway. **(B)** Atopic Dermatitis (AD): In macrophages, ultraviolet-treated riboflavin suppresses histone H3 lysine 9 lactylation (H3K9la) at the promoters of nucleotide-binding oligomerization domain-like receptor family pyrin domain containing 3 (NLRP3) and apoptosis-associated speck-like protein containing a CARD (ASC). This epigenetic suppression inhibits NLRP3 inflammasome activation and M1 macrophage polarization, reducing interleukin-1β (IL-1β) secretion. Consequently, keratinocyte-derived thymic stromal lymphopoietin (TSLP) production is attenuated, alleviating the inflammatory cascade in atopic dermatitis. **(C)** Hypertrophic Scar: In fibroblasts, lactate-derived lactylation of histone H3 lysine 18 (H3K18la) upregulates SLUG expression, which transcriptionally represses phosphatase and tensin homolog (PTEN). PTEN suppression blocks autophagy, leading to increased collagen deposition and fibroblast proliferation. Silencing lactate dehydrogenase A (LDHA) reduces H3K18la, restoring PTEN expression and autophagic activity, thereby reducing scar formation. **(D)** Melanoma: Two interconnected pathways are depicted. In melanoma cells, H3K18la promotes transcription of YTH N6-methyladenosine RNA-binding protein 2 (YTHDF2), which recognizes m6A-modified mRNAs of tumor suppressor genes PER1 and TP53, facilitating their degradation and promoting tumorigenesis. In tumor-associated endothelial cells, lactate enhances H3K18la at the promoter of stromal tumor suppressor 2 (ST2), increasing endothelial cell responsiveness to pro-angiogenic interleukin-33 (IL-33). This promotes vascular proliferation and tumor angiogenesis. IL-17A, interleukin-17A; IL-17R, interleukin-17 receptor; KLK8, kallikrein 8; HAT1, histone acetyltransferase 1; H4K12la, histone H4 lysine 12 lactylation; FOXM1, forkhead box M1; CCNB2, cyclin B2; CDC25C, cell division cycle 25C; MCT1, monocarboxylate transporter 1; AD, atopic dermatitis; H3K9la, histone H3 lysine 9 lactylation; NLRP3, NOD-like receptor family pyrin domain containing 3; ASC, apoptosis-associated speck-like protein containing a CARD; IL-1β, interleukin-1 beta; TSLP, thymic stromal lymphopoietin; H3K18la, histone H3 lysine 18 lactylation; PTEN, phosphatase and tensin homolog; LDHA, lactate dehydrogenase A; YTHDF2, YTH N6-methyladenosine RNA-binding protein 2; PER1, period circadian regulator 1; TP53, tumor protein p53; ST2, stromal tumor suppressor 2 (also known as IL-33 receptor); IL-33, interleukin-33.

Current research indicates a direct association between lactylation and the pathogenesis of psoriasis. Shen X et al. (8) identified a skin tissue-specific IL-17 signalling pathway in psoriasis that operates independently of standard chemokines. IL-17A induces keratinocyte-specific KLK8 to interact with IL-17R, thereby promoting HAT1 to catalyse H4K12la lactylation. H4K12la further upregulates KLK8, IL-17R, and cell cycle-associated genes (e.g., FOXM1, CCNB2, CDC25C), accelerating keratinocyte proliferation and forming a positive feedback loop that exacerbates psoriasis progression (shown in **Figure 3A**). This excessive activation of atypical IL-17A signalling is driven by hyperlactate levels in the microenvironment originating from multiple cellular sources. It diminishes the efficacy of anti-IL-17A therapies for psoriasis. Unexplained reduced responsiveness to biologic treatments frequently occurs in patients with psoriasis and metabolic disorders (e.g., obesity, diabetes mellitus, hypertension) (103–105). Notably, psoriasis patients with metabolic comorbidities typically exhibit elevated lactate concentration in both skin tissue and blood (106–108). The authors administered the MCT1 inhibitor AZD3965 via intraperitoneal injection in combination with subcutaneous injection of anti-mIL-17A antibody to treat psoriasis mice following lactate administration. They observed that AZD3965 significantly improved the efficacy of the anti-mIL-17A antibody impaired by elevated lactate levels. The combined administration of subcutaneous anti-mIL-17A antibody and intraperitoneal 2-DG also effectively suppressed disease progression in psoriasis mice, demonstrating superior efficacy to either monotherapy in reducing PASI scores, skin thickening, and H4K12la expression. This discovery offers a novel therapeutic approach for psoriasis, particularly in patients with obesity, lactate metabolism abnormalities, and metabolic syndrome. Furthermore, combining lactylation inhibition with anti-IL-17A therapy provides a new direction for addressing biologic-resistant psoriasis. Nevertheless, this study retains limitations. Firstly, *in vitro* experiments utilised HaCaT cells exclusively, whose biological characteristics differ from primary keratinocytes. Secondly, clinical evidence primarily relies on retrospective single-cell sequencing and patient sample analyses, lacking prospective cohort validation. Furthermore, the clinical safety and efficacy of lactate inhibitors (such as 2-DG and oxamate) for psoriasis require further investigation.

Lactylation, which links metabolic reprogramming and epigenetic regulation, has shown preliminary evidence of a potential biological role in the pathogenesis and progression of psoriasis. Whether this modification directly mediates the pathological processes of psoriasis or exerts regulatory effects during lesion repair and immune microenvironment remodelling warrants further investigation. Furthermore, prospective studies on lactate metabolism and the extent of lactylation in psoriasis patients, particularly those with concomitant metabolic syndrome, as well as longitudinal clinical investigations examining alterations in lactate metabolism and lactylation levels before and after treatment, are urgently required.

### 5.2 Atopic dermatitis

The pathogenesis of AD is complex. It is currently understood to result from the interaction of genetic and environmental factors. This chronic inflammatory skin condition is mediated by immune pathways and characterised by disruption of the skin barrier and dysbiosis of the cutaneous microbiome. Research indicates that topical application of novel zinc lactate emollients can reduce skin surface pH and improve skin barrier function in patients with AD (109). In a randomised double-blind study involving 50 patients with dry eczema (including AD), two lactate-containing emollients demonstrated good tolerability. They were also significantly associated with improvements in itch severity, skin hydration, and lipid content (110). Furthermore, Lactobacillus can reduce the adhesion of Staphylococcus aureus to keratinocytes and upregulate the expression of several key antimicrobial peptides (AMPs), such as β-defensin 2 (111, 112). Myles IA et al. (113) observed that the topical application of Lactobacillus significantly improved SCORAD scores in patients with AD. The authors posit that patients with AD and more severely disrupted skin microbiomes may exhibit greater responsiveness to Lactobacillus therapy.

While the role of lactate in AD pathophysiology is increasingly recognized, direct evidence for lactylation as a mechanistic driver in AD remains sparse. Metabolic reprogramming may constitute a key driver of the inflammatory microenvironment within AD skin lesions. *In vitro* cellular models of inflammatory dendritic epidermal cells (IDECs) in AD exhibit increased extracellular lactate concentrations, glycolytic rates, and glycolytic capacity. Glycolysis in IDECs may upregulate inflammatory cytokine expression by activating receptor I for the Fc region of immunoglobulin E (FcϵRI) and Toll-like receptor 2 (TLR2) (114). This suggests that danger signals such as bacteria or allergens may stimulate inflammatory responses in AD via the glycolytic pathway. Lactate accumulation resulting from glycolytic upregulation may mediate AD epigenetic regulation by inducing lactylation. Among the limited direct evidence, studies in AD mouse models and cellular research (9) revealed that ultraviolet-treated riboflavin suppresses H3K9la in the nucleotide-binding oligomerisation domain-like receptor family pyrin domain containing 3 (NLRP3) and the apoptosis-associated speck-like protein (Apoptosis-associated Speck-like protein containing a CARD, ASC) to target the NLRP3 inflammasome in macrophages. This epigenetic regulation can simultaneously suppress M1 macrophage polarisation and reduce their secretion of IL-1β and keratinocyte-produced thymic stromal lymphopoietin (TSLP), effectively mitigating the inflammatory cascade in AD and attenuating disease severity (shown in **Figure 3B**). However, this study focused solely on macrophages and keratinocytes, utilising only the MC903-induced AD mouse model without encompassing other allergen-induced models, thus lacking heterogeneity. Further investigation is required into the regulatory role of lactylation in AD-associated keratinocytes, other immune cells, and signalling pathways. Moreover, whether ultraviolet radiation directly alters the lactylation status of AD-associated cells, thereby participating in disease pathogenesis, warrants further investigation.

Lactylation shows potential for research in the treatment of AD. However, currently there is no direct evidence linking it to the pathogenesis of AD. Identifying the specific pathways and key regulatory mechanisms involved in lactylation during AD pathogenesis will aid in the discovery of novel therapeutic targets for the disease.

### 5.3 Hypertrophic scars and keloids

Scar is a natural product of the wound-healing process in human body. Pathologic scars primarily encompass hypertrophic scars and keloids. Recent research indicates that hypertrophic scars and keloids share numerous clinical and histological commonalities (115). They both arise from excessive fibroblast proliferation and collagen deposition following skin injury. Fibroblasts, as primary cells in scar formation, are responsible for synthesising collagen and the extracellular matrix. Excessive collagen deposition and microvascular occlusion create hypoxic microenvironments within pathological scars. Keloids are fibrous tissue tumour exhibiting tumour-like biological characteristics, including the Warburg effect. Studies indicate that *in vitro*-cultured keloid fibroblasts exhibit markedly increased glucose uptake and lactate production, along with upregulation of glycolysis-related enzyme expression. Furthermore, the glycolysis inhibitor 2-deoxyglucose (2-DG) significantly suppresses cellular proliferation (116). Further investigations reveal that under normoxic conditions, glycolysis-related gene and protein expression, as well as enzyme activity, are higher in keloid fibroblasts than in normal skin fibroblasts. Under hypoxic conditions, glycolysis intensifies, mitochondrial function declines, and cellular proliferation, migration, invasion, and collagen synthesis increase, whereas apoptosis decreases (117). Moreover, enhanced glycolysis and lactate accumulation were observed in hypertrophic scar tissue and its fibroblasts, with glycolysis inhibition attenuating fibroblast activation (118). These findings indicate that enhanced glycolytic metabolism in pathological scar fibroblasts depends not only on the hypoxic microenvironment but also reflects an active fibroblast choice for a rapid proliferative metabolic mode. This phenomenon bears similarities to the Warburg effect observed in tumour cells. Intervention in lactate-related metabolic pathways may represent an effective strategy to mitigate excessive fibroblast proliferation within pathological scars.

While the aforementioned studies establish that enhanced glycolysis and lactate accumulation characterize pathological scars, the specific involvement of lactylation in this context has only begun to be explored. Lactate may directly influence fibroblast activation via lactylation, thereby exacerbating excessive fibroblast proliferation within pathological scars. Integrated analysis of lactate-related gene sets and proteome data revealed that 14 lactylation sites were significantly upregulated in hypertrophic scar tissue. Lactylation is associated with ribosomal function and the regulation of glycolysis/gluconeogenesis, potentially contributing to the formation of hypertrophic scars (119). Liu X et al. (10) observed elevated lactylation level in hypertrophic scar tissue and its fibroblasts. *In vitro* studies indicate that H3K18la upregulation promotes collagen deposition and cellular viability in fibroblasts. Meanwhile, silencing LDHA, which is associated with this histone lactylation, can promote PTEN transcription by inhibiting SLUG, thereby enhancing fibroblast autophagy and reducing collagen deposition and cellular proliferative activity (shown in **Figure 3C**). This suggests that targeting transcriptional regulation via lactylation may offer an effective therapeutic strategy for hypertrophic scars. Integrated analysis of lactylated motif and proteomic data identified 14 lactylation sites that were significantly upregulated in hypertrophic scar tissue. Lactylation correlates with ribosomal function and regulation of glycolysis/gluconeogenesis, potentially contributing to hypertrophic scar formation (116). However, this study remains confined to HSFs and lacks validation through animal models. Furthermore, the clinical sample size is too small and lacks healthy controls, limiting the generalisability of its findings. Additionally, the key sites where histone lactylation regulates SLUG warrant further investigation.

Although the molecular network governing lactylation-mediated regulation of pathological scar formation remains incompletely elucidated, preliminary studies have revealed its role in regulating fibroblast activation and collagen deposition. Future research should focus on identifying potential regulatory sites and key targets of lactylation, thereby providing novel insights for clinical interventions targeting pathological scars.

### 5.4 Melanoma

Melanoma is the most malignant form of skin tumour. Its characteristics include complex metabolic reprogramming, immune evasion, and angiogenesis. Most melanoma cells exhibit the Warburg effect. Compared to normal melanocytes, they demonstrate markedly increased glucose uptake and lactate production (120). Lactate is a pivotal factor in shaping the tumour microenvironment (121). In melanoma, dysregulated glycolysis leads to excessive lactate production and tumour microenvironment acidification, thereby driving tumour growth, invasion, and therapeutic resistance (122). Concurrently, lactate suppresses cytotoxic T-cell function and polarises macrophages towards a pro-tumour M2 phenotype (123). Induction of angiogenesis is also widely recognised as an essential process during the progression of most human tumours, including melanoma (124). It has been reported that lactate also plays a key role in promoting tumour angiogenesis. Research by De Saedeleer CJ et al. (125) in human tumour cell lines indicates that, lactate produced by tumour cells can be taken up by vascular endothelial cells, where it interacts with the GPR81 receptor to activate hypoxia-inducible factor 1 (HIF-1), thereby increasing vascular endothelial growth factor (VEGF) expression. This process coordinates endothelial cell proliferation and migration programmes, promoting angiogenesis. The role of lactate as a signalling molecule in angiogenesis even surpasses its role in metabolism (126).

Accumulating evidence indicates that lactate and lactylation represent a crossroads between metabolic reprogramming and immune suppression in tumours (127). Lactylation-associated genes have emerged as significant biomarkers for multiple malignancies, including melanoma (128–130). Yu J et al. (11) demonstrated that ocular melanoma tissue exhibits significantly elevated overall lactylation and H3K18la level. This correlates with poor prognosis in patients. Inhibiting lactylation suppresses the progression of ocular melanoma. In human ocular melanoma cell lines (92.1, MUM2B and OCM1), H3K18la promotes transcription of YTH N6-methyladenosine RNA-binding protein 2 (YTHDF2). This protein recognises m6A modification sites on the PER1 and TP53 tumour suppressor genes. It facilitates their degradation, thereby promoting tumorigenesis (shown in **Figure 3D**). Concurrently, treatment with glycolysis inhibitors (2-DG and oxmate) significantly reduced intracellular lactate levels, overall lactylation, and H3K18la levels in ocular melanoma cells. The decrease in lactylation levels also effectively inhibited the proliferation of ocular melanoma cells. This study links histone modifications with RNA modifications, offering novel insights into epigenetic regulation. It further highlights the correlation between histone lactylation levels and prognosis in ocular melanoma patients. Collectively, these findings suggest lactylation may serve as a prognostic indicator for melanoma, potentially correlating with tumour staging. Wang L et al. (75) reported that high-risk melanoma exhibits elevated M2 macrophage infiltration and CD8+ T cell exhaustion. And lactylation was a novel regulator of this immunosuppressive shift. Previous studies indicate that macrophage migration inhibitory factor (MIF) secreted by breast cancer tumour cells binds to CD74 on macrophages, activating CXCR4-dependent PI3K/AKT signalling. This stabilises HIF-1α and further amplifies glycolysis in tumour-associated macrophages, forming a vicious cycle that sustains lactate production (131). Lactate accumulation in melanoma may polarise macrophages towards an M2 phenotype via lactylation, thereby sustaining MIF signalling to enhance immune evasion.

Furthermore, Zhao M et al. (12) demonstrated that lactate and lactylation promote melanoma angiogenesis *in vivo* and *in vitro*. Lactate enhances transcription of stromal tumour suppressor 2 (ST2) in tumour-associated endothelial cells by amplifying H3K18la at its promoter. This increases endothelial cell responsiveness to proangiogenic interleukin-33 (IL-33) stimulation, thereby promoting vascular proliferation (shown in **Figure 3D**). Lactate also inhibits the high endothelial venular transition of endothelial cells, which is critical for tumour development. Anti-angiogenic drugs synergistically inhibit melanoma angiogenesis and *in vivo* growth when combined with lactate dehydrogenase (LDH) inhibitors/ST2 inhibitors. This discovery of targeting lactylation provides a compelling rationale for developing combination therapies against drug-resistant melanoma.

Lactylation promotes tumour cell proliferation, invasion, immune evasion, and angiogenesis through immune suppression, metabolic reprogramming, and gene expression regulation. It thereby contributes to melanoma progression. Targeting immune suppression mediated by lactylation and rebalancing the tumour microenvironment via epigenetic modifications holds promise for personalised melanoma treatment.

### 5.5 Skin ageing

Skin ageing results from the combined influence of endogenous factors (genetics, age, hormonal levels, etc.) and exogenous factors (ultraviolet radiation, environmental pollution, poor lifestyle habits, etc.). Its primary characteristic is a reduction in collagen synthesis within fibroblasts. Lactate can stimulate the expression of hyaluronic acid and CD44 (the primary receptor for hyaluronic acid) in fibroblasts (132). Currently, polylactate has been shown to alleviate skin ageing. Poly-D, L-lactic acid (PDLLA) stimulates collagen synthesis and promotes angiogenesis in aged mouse models (133). Furthermore, lactate released from PLLA can promote K752 lactylation of LTBP1, and subsequently increase type I and type III collagen levels in fibroblasts (43). This indicates that lactylation can mediate lactate’s pro-collagen effects. However, whether endogenous lactylation contributes to physiological or pathological skin ageing remains unknown. Furthermore, whether metabolic-related diseases induce skin intrinsic ageing through lactate and lactylation, and whether ultraviolet radiation participates in skin extrinsic ageing processes by regulating lactylation, also warrants further investigation.

### 5.6 Hair growth

Lactate and its associated metabolic pathways play a pivotal role in hair growth, closely linked to the highly metabolic nature of hair follicles. Hair follicle stem cells, as key players in hair development, exhibit active glycolytic metabolism. Even under aerobic conditions, they preferentially generate energy through glycolysis, leading to persistently elevated local lactate levels within the follicle (134). Lactate can promote the production of VEGF, stimulating angiogenesis around hair follicles by acting on dermal vascular endothelial cells. This nourishes the hair follicles and promotes hair growth (135, 136). Deficiency in lactate and LDH-related genes inhibits hair follicle stem cell activation, whereas lactate induction accelerates their activation and the hair cycle (84). Although no definitive evidence currently exists demonstrating a direct correlation between lactylation and hair growth, extensive research indicates that lactylation, as a key lactate-mediated epigenetic regulatory mechanism, participates in cell cycle regulation and processes such as stem cell proliferation, differentiation, and apoptosis (88, 137, 138). As metabolically active cells, the proliferation, differentiation, and cell cycle of hair follicle cells are intrinsically linked to the hair cycle. By analogy, lactate metabolism during hair follicular development might theoretically influence the follicular cycle via lactylation, but this hypothesis awaits direct experimental testing in hair biology models.

## 6 Potential therapeutic targets and drugs based on lactylation

### 6.1 Regulating the process of lactate production

Currently, therapies targeting lactylation primarily focus on regulating lactate production. And some treatments have progressed to preclinical or early clinical stages. Glycolysis inhibitors have demonstrated efficacy in cancer treatment by suppressing lactate production and reducing lactylation (139). Among these, 2-DG, a synthetic glucose analogue, accumulates intracellularly and blocks glycolysis by inhibiting hexokinase. In psoriasis mouse models, 2-DG reduces H4K12la levels and alleviates epidermal hyperplasia. When combined with anti-IL-17A antibodies, it significantly enhances efficacy against psoriasis associated with metabolic syndrome (8). Oxamate, as a specific inhibitor of LDHA, can block the conversion of pyruvate to lactate in the glycolytic pathway. It regulates cellular metabolism, the immune microenvironment, and tumour malignant phenotypes. By inhibiting LDHA, oxamate demonstrates broad therapeutic potential with notable effects across cancer, metabolic, and immunological domains. It suppresses tumour growth by inhibiting glycolysis in cancer cells and enhances their sensitivity to radiotherapy and immunotherapy (140–142). For metabolic disorders, it markedly improves glycaemic control and insulin sensitivity. When used in combination with metformin, it markedly reduces levels of inflammatory markers (143). Its advantages include low cytotoxicity towards normal cells and the potential to minimise systemic side effects through local administration (e.g., gels, injectables). Mechanistically, oxamate inhibits LDHA, reducing lactate production and disrupting cellular metabolism. This inhibits abnormal cell proliferation, regulates metabolic function, and enhances the efficacy of immunotherapy. Given the hallmark features of keratinocyte hyperplasia and metabolic dysfunction in psoriasis, oxamate demonstrates therapeutic potential for this condition. Fargesin, a novel lignan isolated from Magnoliaceae plants, exhibits antitumour activity. Research indicates that in non-small cell lung cancer, it targets the glycolytic rate-limiting enzyme pyruvate kinase 2 (PKM2). By inhibiting histone H3 lactylation, it suppresses tumour growth (144). Abnormal glycolysis constitutes a core metabolic hallmark of skin tumours. The distinct targeted regulatory mechanism of fargesin on the glycolytic pathway (targeting PKM2) suggests its potential for treating a broad range of malignancies characterized by abnormal glycolysis, including skin tumours.

### 6.2 Targeting specific lactylation sites

Targeting specific lactylation sites can also enable precise intervention in lactylation. Royal jelly acid is a natural compound that interferes with lactate production. In human hepatocellular carcinoma cell lines, it inhibits cell proliferation and migration by suppressing lactylation at the H3K9 and H3K14 sites, whilst significantly inhibiting tumour growth in subcutaneous tumours in nude mice (145). Demethylzeylasteral (DML), a triterpenoid antitumour compound, similarly inhibits the proliferation and migration of liver cancer stem cells whilst inducing apoptosis. Its antitumour effects primarily arise through suppression of lactylation at H3K9la and H3K56la sites (21). In prostate cancer, evodiamine significantly blocks lactate-induced angiogenesis and inhibits tumour progression by suppressing H3K18la and HIF1α expression in prostate cancer cells (146). This demonstrates that the aforementioned natural compounds, which target specific lactylation sites, function as precise modulators of lactylation. They play a pivotal role in anti-angiogenesis and immune regulation. This offers novel therapeutic approaches for skin tumours and vascular skin diseases, demonstrating broad application prospects.

### 6.3 Targeting enzymes involved in the lactylation process

During the process of lactylation, lactyltransferases and deacetylases also play crucial roles. As ‘erasers’ of lactylation, SIRT family deacetylases mediate the removal of lactyl groups (2, 3). For instance, SIRT3 has been demonstrated to possess delactylase activity. Honokiol (HKL), by activating SIRT3, regulates the lactylation level of CCNE2, thereby inducing cancer cell apoptosis. This mechanism not only inhibits cell proliferation but also reduces hepatic damage (147). In skin biology, SIRT3 is involved in keratinocyte differentiation, UV responses, and melanoma progression (148); while SIRT1 is downregulated in psoriasis, suggesting its’ anti-proliferative role (149). Despite these findings, direct evidence linking lactyltransferases and delactylases to specific skin diseases remains limited, representing a current gap in the field. Future studies are needed to clarify their roles in abnormal keratinocyte proliferation, inflammatory imbalance, and fibrotic processes.

### 6.4 Lactylation combined with immunotherapy

Currently, selective targeted immunotherapy has achieved significant advances across multiple dermatological conditions. However, an increasing number of studies indicate that lactate-driven lactylation impedes the efficacy of immunotherapy (150). By inhibiting lactate production to reduce lactylation, thereby alleviating its suppression of immunotherapy, the overall therapeutic efficacy can be enhanced. This combined treatment regimen may offer a novel approach to addressing immunotherapy resistance in skin diseases such as psoriasis and AD.

### 6.5 Clinical translation challenges and future directions

While preclinical studies have identified promising lactylation-targeted interventions, clinical translation requires careful consideration of specificity, safety, and delivery modalities (**Table 1**). The only FDA-approved HDAC inhibitors for skin diseases (vorinostat and romidepsin for CTCL) are administered systemically and carry significant off-target toxicities. Remetinostat represents a paradigm shift as a topical HDAC inhibitor with negligible systemic exposure, demonstrating that local epigenetic modulation is feasible in dermatology. However, no clinical trial to date has specifically targeted lactylation as a primary mechanism of action. Future clinical development should prioritize: (i) topical formulations to minimize systemic exposure; (ii) site-selective lactylation modulators (e.g., H3K18la- or H4K12la-specific); and (iii) biomarker-driven trial designs incorporating skin lactylation levels as pharmacodynamic endpoints.

**Table 1:** Clinical trials of lactate metabolism and lactylation-targeted interventions in skin diseases and related conditions.

Agent	Target/mechanism	Indication	ClinicalTrials.gov identifier	Phase	Key findings/status	Specificity & safety considerations	Topical delivery	Off-target risks
Remetinostat (SHAPE)	Pan-HDAC inhibitor (metabolically labile ester bond design)	Basal cell carcinoma (BCC); Cutaneous T-cell lymphoma (CTCL)	NCT03180528; NCT02213861	Phase 2 (completed)	Topical 1% gel TID for 6 weeks achieved ≥30% tumor reduction in BCC; well-tolerated in CTCL with no systemic HDAC inhibitor-related adverse events	High skin specificity: Rapid metabolism in blood minimizes systemic exposure (plasma levels below quantification limit); primarily local skin irritation/pruritus	Yes (topical gel formulation)	Minimal systemic toxicity due to metabolic instability; local skin reactions only
Vorinostat (SAHA)	Pan-HDAC inhibitor (Classes I, II, IV)	Cutaneous T-cell lymphoma (CTCL)	NCT00050800; NCT00419367 (compassionate use)	FDA-approved (2006)	ORR ~30% in refractory CTCL; improved skin lesions and pruritus; pivotal trials established efficacy	Low specificity: Systemic administration affects multiple tissues; broad HDAC inhibition	No (oral)	Fatigue, diarrhoea, thrombocytopenia, nausea; cardiac QT prolongation risk
Romidepsin	Class I HDAC inhibitor (HDAC1, 2, 3)	Cutaneous T-cell lymphoma (CTCL); Peripheral T-cell lymphoma (PTCL)	NCT00337025; NCT01445340 (topical)	FDA-approved (2009)	ORR 34–38% in CTCL; median duration of response ~15 months; intravenous administration	Moderate specificity: Class I-selective but systemic delivery; cardiac toxicity monitoring required	No (IV); topical formulation under investigation (NCT01445340)	Cardiac toxicity (QT prolongation), myelosuppression, infections
Belinostat	Pan-HDAC inhibitor (Classes I, II, IV)	Peripheral T-cell lymphoma (PTCL); Adult T-cell leukaemia/lymphoma (ATLL) with skin involvement	NCT02737046	FDA-approved (2014)	ORR 26% in PTCL; manageable safety profile; thrombocytopenia and neutropenia most common Grade 3/4 AEs	Low specificity: Systemic pan-HDAC inhibition; IV infusion required	No (IV infusion)	Hematologic toxicity, fatigue, nausea; potential for hepatic dysfunction
SRT2104	SIRT1 activator (delactylase enhancer)	Moderate-to-severe plaque psoriasis	NCT01154101	Phase 2 (completed)	35% of patients showed significant histological improvement; downregulated TNF-α and IL-17 signaling; dose-dependent PASI improvement trend; favourable safety profile	Moderate specificity: Oral SIRT1 activation affects multiple tissues; variable interpatient pharmacokinetics (AUC CV 51–89%)	No (oral)	Generally well-tolerated; mild dizziness, headache, upper respiratory infections
Metformin	Mitochondrial complex I inhibitor (indirect glycolysis/lactate reduction)	Chronic plaque psoriasis	NCT02644954	Phase 2	Evaluated as adjunctive therapy; leverages anti-inflammatory and metabolic-modulating properties; ongoing investigation in psoriatic patients with metabolic syndrome	Low specificity: Systemic metabolic effects; gastrointestinal disturbances common	No (oral)	Gastrointestinal adverse events; rare lactic acidosis risk; vitamin B12 deficiency
AZD3965	MCT1 inhibitor (blocks lactate export)	Advanced solid tumors; Diffuse large B-cell lymphoma (DLBCL); Burkitt lymphoma	NCT01791595	Phase 1 (completed)	First-in-class MCT1 inhibitor; dose-limiting toxicities included hyperlactatemia (>8 mmol/L) and metabolic acidosis; limited efficacy in solid tumors	Low specificity: Systemic MCT1 inhibition disrupts normal lactate homeostasis; retinal toxicity observed in preclinical models	No (oral capsules)	Hyperlactatemia, metabolic acidosis, QTc prolongation risk; narrow therapeutic window
2-Deoxyglucose (2-DG)	Hexokinase inhibitor (glycolysis blockade)	Advanced cancer; Hormone-refractory prostate cancer	NCT00096707; NCT00633087	Phase 1/2	Limited clinical development due to poor tolerability; systemic hypoglycaemia and neurotoxicity concerns; preclinical efficacy in psoriasis models (H4K12la reduction)	Very low specificity: Essential glycolysis inhibition affects all tissues; narrow therapeutic index	No (oral/IV)	Hypoglycaemia, neurotoxicity, nausea, fatigue; unacceptable toxicity profile for dermatological use
Panobinostat	Pan-HDAC inhibitor (Classes I, II, IV)	Multiple myeloma; GVHD prevention (skin involvement)	NCT02588339; NCT01065446	FDA-approved (2015) for multiple myeloma	Demonstrated efficacy in hematologic malignancies; immunomodulatory properties relevant to skin inflammation; significant toxicity profile	Low specificity: Systemic pan-HDAC inhibition with broad epigenetic effects	No (oral)	Severe diarrhoea, fatigue, thrombocytopenia, cardiac toxicity; not suitable for localized skin disease
Entinostat (MS-275)	Class I-selective HDAC inhibitor (HDAC1, 3)	Solid tumors; Combination with immunotherapy	NCT03215264; NCT03552380	Phase 1/2	Long half-life (~50 hours); favourable side effect profile compared to pan-HDAC inhibitors; Treg suppression and immune enhancement demonstrated	Moderate specificity: Class I-selective reduces but does not eliminate off-target effects; oral bioavailability	No (oral)	Fatigue, nausea, myelosuppression; hypophosphatemia and electrolyte abnormalities

## 7 Conclusion

In summary, lactylation reveals the pivotal role of metabolic reprogramming and epigenetic regulation in the pathogenesis of skin diseases. Through a ‘metabolite-epigenetic-immune’ axis, lactylation modulates important pathophysiological processes in the skin, including inflammatory responses, abnormal cell proliferation, and tissue remodelling. Research indicates that dysregulated lactylation, driven by elevated lactate levels, significantly accelerates the progression of immune−inflammatory skin disorders (such as psoriasis and atopic dermatitis). It may also contribute to pathological scar formation, skin ageing, hair growth, and the development of skin tumours, including melanoma (**Table 2**), although the underlying mechanisms remain to be further investigated. Lactylation is a double-edged sword, activating reparative or pro-inflammatory genes by modifying histones and altering enzyme function through modifying non-histone proteins. Its effects are largely influenced by specific disease types and the immune microenvironment (151, 152). However, it is important to acknowledge the current boundaries. While lactate’s roles in skin physiology and pathology are well-documented, the field of lactylation in dermatology remains in its infancy. Many of the connections drawn in this review between lactate metabolism and disease mechanisms are extrapolations that await direct validation through site-specific lactylation mapping, genetic or pharmacological manipulation of lactylation enzymes (‘Writers’ and ‘Erasers’), and disease models with targeted lactylation reporters.

**Table 2:** Overview of lactylation in skin diseases and physiological conditions.

Disease-related	Lactylprotein	Expression level	Regulatory target	Reference
Psoriasis	H4K12la	↑	KLK8, HAT1, IL-17	(10)
Atopic Dermatitis (AD) alleviation	H3K9la	↓	NLRP3, IL-1β, TSLP	(11)
Hypertrophic scar	H3K18la	↑	LDHA, SLUG, PTEN	(12)
Melanoma (oncogenesis)	H3K18la	↑	YTHDF2, PER1, TP53 RNA	(13)
Melanoma (angiogenesis)	H3K18la	↑	ST2, IL-33	(14)
Skin ageing retardation	K752la	↑	LTBP1, KAT8	(66)

Arrows indicate upregulation (↑) or downregulation (↓) of lactylation. This table includes only diseases where lactylation has been directly demonstrated at specific histone or non-histone residues. For conditions such as skin ageing and hair growth, where lactate roles are established but direct lactylation evidence is lacking, see text for discussion of hypothetical mechanisms. H4K12la, histone H4 lysine 12 lactylation; KLK8, kallikrein 8; HAT1, histone acetyltransferase 1; IL-17, interleukin-17; H3K9la, histone H3 lysine 9 lactylation; AD, atopic dermatitis; NLRP3, NOD-like receptor family pyrin domain containing 3; IL-1β, interleukin-1 beta; TSLP, thymic stromal lymphopoietin; H3K18la, histone H3 lysine 18 lactylation; LDHA, lactate dehydrogenase A; PTEN, phosphatase and tensin homolog; YTHDF2, YTH N6-methyladenosine RNA-binding protein 2; PER1, period circadian regulator 1; TP53, tumor protein p53; ST2, stromal tumor suppressor 2 (IL-33 receptor); IL-33, interleukin-33; K752la, lysine 752 lactylation; LTBP1, latent transforming growth factor-β binding protein 1; KAT8, lysine acetyltransferase 8.

Furthermore, lactate−induced lactylation may similarly play a pivotal role in other skin diseases. For example, similar lactylation modifications observed in cardiovascular diseases may also occur in endothelial cells of cutaneous vasculitis. Lactylation can drive the transformation of endothelial cells into mesenchymal cells, leading to increased vascular permeability and inflammatory responses (153). In inflammatory skin diseases involving the sebaceous glands, such as acne, rosacea, and seborrhoeic dermatitis, the hypoxic and lipid-rich microenvironment may promote glycolysis in neutrophils and macrophages. The resulting accumulation of lactate may induce lactylation, upregulate pro-inflammatory cytokine expression, and lead to the persistence of chronic inflammation (154). Moreover, in fungal skin disease, lactylation may exert a dual role. It can both modulate the host immune response and regulate the energy metabolism and gene expression of the pathogen, thereby influencing the virulence and drug resistance of the pathogen (155, 156).

The therapeutic potential of targeting lactylation is increasingly evident. By modulating glycolysis, inhibiting lactate production, or directly targeting enzymes involved in lactylation, novel approaches have been identified for restoring normal metabolism and immune homeostasis in skin tissue. By acting on upstream metabolic drivers of disease, such interventions hold promise as safer and more effective alternatives to existing treatments.

Nevertheless, translating these findings into clinical practice presents several challenges. Firstly, it is crucial to clarify the strength of association between lactylation and the onset and progression of skin diseases, as well as its impact on therapeutic response. Secondly, the epigenetic regulatory mechanisms of lactylation across different skin conditions should be elucidated. While some universal regulators of lactylation have been identified, skin-specific lactylation-associated enzymes and their precise targets require further investigation. Moreover, the protective versus detrimental roles of lactylation in acute versus chronic skin inflammation remain to be elucidated. Finally, future efforts should focus on identifying specific lactylation biomarkers in skin samples and on developing highly selective small-molecule inhibitors or agonists. A deeper understanding of these molecular mechanisms will undoubtedly open new avenues for precision therapies in refractory skin diseases.

## Glossary

2-DG: 2-Deoxyglucose
AD: Atopic Dermatitis
AKT: Protein Kinase B
ALDOA: Aldolase A
AMPs: Antimicrobial Peptides
anti-mIL-17A: Anti-Mouse Interleukin-17A
AURKA: Aurora Kinase A
ASC: Apoptosis-associated Speck-like protein containing a CARD
AZT3965: MCT1 Inhibitor AZT 3965 (commonly known as AZD3965)
CAR-T: Chimeric Antigen Receptor T-cell
CBP: CREB Binding Protein
CCND1: Cyclin D1
CCNE2: Cyclin E2
CCR8: C-C Motif Chemokine Receptor 8
CDC25C: Cell Division Cycle 25C
CD3: Cluster of Differentiation 3
CD4: Cluster of Differentiation 4
CD8: Cluster of Differentiation 8
CD28: Cluster of Differentiation 28
CD44: Cluster of Differentiation 44
CD74: Cluster of Differentiation 74
CoA: Coenzyme A
CXCR4: C-X-C Motif Chemokine Receptor 4
DML: Demethylzeylasteral
DNA: Deoxyribonucleic Acid
FcϵRI: Receptor I for the Fc region of Immunoglobulin E
FOXM1: Forkhead Box M1
Foxp3: Forkhead Box P3
GLUT: Glucose Transporter
GRP81: G Protein-Coupled Receptor 81
HAT1: Histone Acetyltransferase 1
HATs: Histone Acetyltransferases
HCC: Hepatocellular Carcinoma
HIF1-α: Hypoxia-Inducible Factor 1-alpha
HKL: Honokiol
HMGB1: High Mobility Group Box 1
HMP: Hexose Monophosphate Pathway
HDACs: Histone Deacetylases
IDECs: Inflammatory Dendritic Epidermal Cells
IgE: Immunoglobulin E
IR780: A near-infrared fluorescent dye
KAT8: Lysine Acetyltransferase 8/Histone Acetyltransferase KAT8
KLK8: Kallikrein-related Peptidase 8
Lac-CoA: Lactyl-Coenzyme A
LDH: Lactate Dehydrogenase
LDHA: Lactate Dehydrogenase A
LPS: Lipopolysaccharide
LTBP1: Latent Transforming Growth Factor-β
MAPK: Mitogen-Activated Protein Kinase
MC903: Calcipotriol (a vitamin D3 analogue)
MCTs: Monocarboxylate Transporters
MIF: Macrophage Migration Inhibitory Factor
MOESIN: Membrane-Organizing Extension Spike Protein
MYC: MYC Proto-Oncogene
m6A: N6-Methyladenosine
mRNA: Messenger RNA
NAD+: Nicotinamide Adenine Dinucleotide (Oxidized)
NADH: Reduced Nicotinamide Adenine Dinucleotide
NF-κB: Nuclear Factor Kappa-light-chain-enhancer of activated B cells
NFκB1: Nuclear Factor Kappa B Subunit 1
NLRP3: Nucleotide-binding Oligomerisation Domain-like Receptor family Pyrin Domain containing 3
NSCLC: Non-Small Cell Lung Cancer
PASI: Psoriasis Area and Severity Index
PD-1: Programmed Cell Death Protein 1
PDLLA: Poly-D,L-Lactic Acid
PER1: Period Circadian Regulator 1
PI3K: Phosphoinositide 3-Kinase
PKM2: Pyruvate Kinase M2
PLLA: Poly-L-Lactic Acid
PP1: Protein Phosphatase 1
PTEN: Phosphatase and Tensin Homologue
PTMs: Post-Translational Modifications
RELA: RELA Proto-Oncogene, NF-κB Subunit RELA
RNA: Ribonucleic Acid
SCORAD: SCORing Atopic Dermatitis
SIRT: Sirtuin
SLUG: Snail Family Transcriptional Repressor 2
ST2: Stromal Tumour Suppressor 2 (Interleukin-33 Receptor)
TCA: Tricarboxylic Acid
TCR: T Cell Receptor
TGF-β: Transforming Growth Factor-Beta
Th17: T Helper 17 Cells
TME: Tumour Microenvironment
TNF-α: Tumour Necrosis Factor-alpha
TP53: Tumour Protein P53
TPX2: Targeting Protein for Xklp2
Treg: Regulatory T Cells
TSLP: Thymic Stromal Lymphopoietin
UV: Ultraviolet
VEGF: Vascular Endothelial Growth Factor
YTHDF2: YTH N6-Methyladenosine RNA-binding Protein 2.
